# Effect of a Dihydroxyacetone‐Based Camouflage Agent on Ultraviolet‐Induced Erythema

**DOI:** 10.1111/jocd.70587

**Published:** 2025-12-05

**Authors:** Dan Shu, Yiting Han, Dian Chen, Zhenghui Li, Yi Zhao

**Affiliations:** ^1^ Department of Dermatology Beijing Tsinghua Changgung Hospital, Tsinghua University Beijing P.R. China; ^2^ School of Clinical Medicine Tsinghua University Beijing China

**Keywords:** camouflage, dihydroxyacetone, erythema, ultraviolet

## Abstract

**Background:**

Vitiligo is an acquired, idiopathic skin disease of dyspigmentation, affecting approximately 0.5%–2% of the global population. However, it is not clear whether dihydroxyacetone (DHA)‐based camouflage could affect the effectiveness of ultraviolet (UV) therapy. Therefore, we aimed to investigate the effect of a camouflage agent containing 3.6% DHA on UV‐induced erythema.

**Methods:**

Fifty‐one healthy volunteers of skin type III to IV who came to the hospital were recruited in this study. A 308 nm excimer UV lamp was used to perform the minimum erythema dose (MED) test on the lower back and a camouflage agent containing 3.6% DHA was applied. Four groups were tested on the same individuals (subjects served as their own controls), including no camouflage nor irradiation (Group A), irradiation without camouflage (Group B), camouflage without irradiation (Group C), and camouflage plus irradiation (Group D). Refined MED was evaluated via naked eyes (erMED) and digital dermatoscopes (drMED), The erythema index (EI) value of the targeted site was measured by dermatoscopic digital imaging and analyzed.

**Results:**

After the camouflage masking, 43 (84.3%) subjects remained unchanged in erMED, 7 (13.7%) had a MED increase of one grade (+10 mJ/cm^2^), and only one (2.0%) had a MED increase of two grades (+20 mJ/cm^2^). Although statistically significant differences were observed in MED (*p* = 0.0047) and EI values (*p* = 0.0015) between groups with and without camouflage, these differences were clinically minimal. No significant difference was observed between erMED and drMED in Group B (*p* = 0.157) or D (*p* = 0.317).

**Conclusion:**

The camouflage agent containing 3.6% DHA exhibits statistically significant but clinically negligible effects on erythema in volunteers exposed to 308 nm UV light, requiring no dose adjustment in 84.3% of cases.

## 1 Introduction

Vitiligo is an acquired, idiopathic skin disease of dyspigmentation, affecting approximately 0.5%–2% of the global population [1]. Characterized by localized depigmentation, the lesions can develop on both the skin and mucous membrane. The combination of topical glucocorticoids and calcineurin inhibitors with phototherapy has been demonstrated to be effective in treating vitiligo on the head and torso [2]. However, patients with acral vitiligo often show unsatisfactory responses to topical treatment. The use of 308 nm excimer phototherapy has the advantage of quick response, short treatment period, good compliance, minimal side effects, and low cumulative dosage. Therefore, it is recognized as one of the first‐line treatment options for vitiligo [3, 4]. The vitiligo lesions on the extremities often cause significant psychological distress in patients, who are often observed to cover the lesions with fabric and/or other camouflage products.

A 3.6% concentration of dihydroxyacetone (DHA) is widely used in chemical camouflage agents [5]. DHA can react with epidermal keratin to form melanoidins, a skin‐color chromophore [6], which shares physicochemical properties with naturally occurring melanin. The camouflage agent becomes effective within 6–8 h, providing uniform coverage of depigmented areas and creating a safe, durable, and waterproof skin‐colored barrier that persists for 7–15 days [7]. However, it is not clear whether DHA‐containing camouflage agents affect the effectiveness of ultraviolet (UV) therapy. In this study, we examined the responses of normal human subjects to 308 nm excimer light, both with and without the application of a long‐lasting camouflage agent containing DHA, to improve the UV therapy protocol for patients currently using chemical camouflages.

## 2 Subjects and Methods

### 2.1 Subjects

**FIGURE 1:**
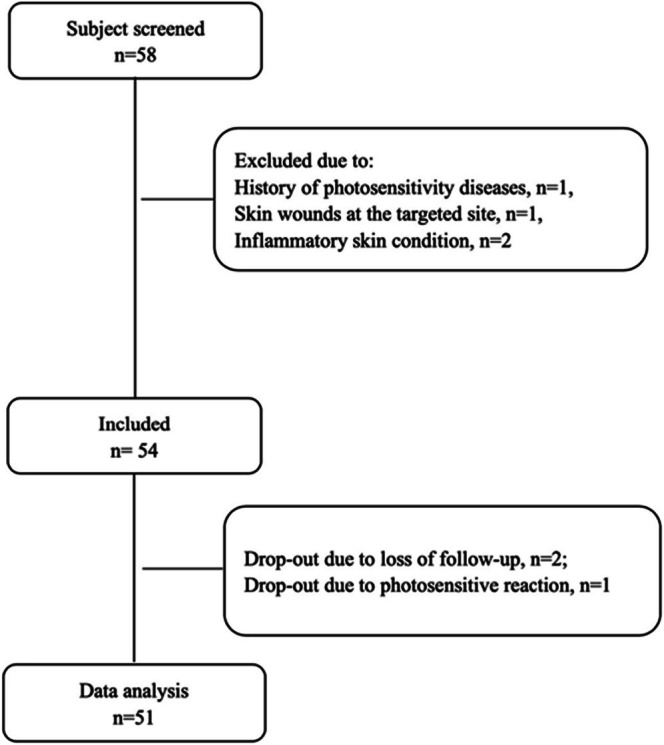
Flow chart of patient selection.

This study was a single‐center, prospective research study, and it was approved by the Ethics Committee. All subjects signed informed consent forms. Healthy subjects who came to the Department of Dermatology of the Hospital from May 23 to June 13, 2018, were included in this study. Inclusion criteria: (1) subjects aged 18–65 years; (2) skin type III to IV; (3) subjects provided informed consent and accepted this treatment plan. Exclusion criteria: (1) subjects had a history of photosensitive diseases; (2) had any current or historical skin cancer; (3) were receiving systemic corticosteroids; (4) were receiving radiotherapy treatment; (5) wounds and inflammatory conditions at tested sites. The flow chart is shown in Figure 1.

### 2.2 Study Instruments and Camouflage Agent

A VTRAC 308 nm excimer light lamp (PhotoMedex Inc., US) was used as the light source. Output dose was 50–4500 mJ/cm^2^, and irradiance was greater than or equal to 150 mW/cm^2^ with 18.9 cm^2^ (6.1 × 3.1 cm) spot size, 3 mm of irradiation distance and 308 ± 2 nm of output wavelength. GaiBailin camouflage liquid containing 3.6% DHA (Beijing Sanheli Biotechnology Co. Ltd) was used as the camouflage agent.

### 2.3 Questionnaire

A questionnaire was designed based on Fitzpatrick skin types [8]. The subjects were allowed to answer the questionnaire under the guidance of the investigator and the skin phototype was recorded by the investigator.

### 2.4 Crude Minimum Erythema Dose (cMED) Test

The healthy skin of the lower back of the subject was irradiated with the 308 nm excimer UV lamp. The skin was divided into eight testing spots in two rows, which were irradiated with a fluence of 50, 70, 90, 110, 130, 150, 170, and 200 mJ/cm^2^, respectively. After 24 h, if the erythema at the irradiation sites was barely visible, well‐defined erythema to the naked eye, the corresponding irradiation flux was determined as cMED. Standardized photographs were taken and archived before irradiation and during each visit. If no erythema was observed at all eight spots after 24 h, a second cMED test was performed with an increased dose series of radiations (Figure S1).

### 2.5 Refined MED Test (rMED) and the Effect of Camouflage on UV‐Induced Erythema

The manually set irradiation flux for a given subject was 20 mJ/cm^2^ lower than the previously measured cMED, with the dose gradually increased by 10 mJ/cm^2^ until cMED was reached. For example, if the subject's cMED was 110 mJ/cm^2^, doses of 90, 100, and 110 mJ/cm^2^ were tested. Three 4 × 4 cm squares were outlined before irradiation, with each square equally divided into four sections (Figure S2). Each section corresponded to a group, including Group A (no camouflage or irradiation), Group B (irradiation without camouflage), Group C (camouflage without irradiation), and Group D (camouflage with irradiation). All four experimental conditions (Groups A, B, C, D) were tested on adjacent skin areas of the same subject to ensure consistency of experimental conditions and comparability of results, with subjects serving as their own controls.

Sites of Groups C and D were evenly coated with the camouflage solution 24 h before irradiation. This 24‐h interval was chosen to simulate steady‐state clinical conditions based on DHA pharmacokinetics: DHA‐keratin reactions complete within 6–8 h, reaching maximum color intensity by 24 h and forming stable melanoidins that persist for 7–15 days without further chemical modification. Therefore, testing at 24 h post‐application effectively simulates the stable camouflage presence that patients experience during ongoing phototherapy sessions, rather than the initial application phase. This design reflects real clinical scenarios where patients present for phototherapy with established camouflage coverage that remains firmly bound to epidermal keratin and is difficult to remove completely, even with regular cleansing.

During irradiation, the sites in Groups A and C were covered with a plastic whiteboard (3 mm thick), allowing only Groups B and D to receive irradiation. After 24 h, erythema was assessed using both naked eye evaluation (erMED) and a digital dermatoscope in noncontact mode (drMED). Finally, the irradiation area displayed in the dermatoscope images was analyzed for erythema index (EI) using the previously described method with Image J software [9, 10].

### 2.6 Statistical Analysis

Statistical analysis was performed using Stata 12.0 (StataCorp., College Station, TX, USA). All data were carefully checked for consistency and plausibility to ensure quality control.

#### 2.6.1 Definition of Study Endpoints

This study employed a hierarchical endpoint structure to systematically evaluate the effect of DHA camouflage on UV‐induced erythema:

##### 2.6.1.1 Primary Endpoint

Change in refined minimum erythema dose assessed by visual examination (erMED), comparing Group B (irradiation without camouflage) versus Group D (irradiation with camouflage). This was selected as the primary endpoint because clinical MED determination by experienced observers represents the gold standard for phototherapy dosing decisions in routine practice.

##### 2.6.1.2 Secondary Endpoints

1. Change in refined minimum erythema dose assessed by digital dermatoscopy (drMED) between Groups B and D, to validate the reliability of visual MED assessment using an objective imaging method.
2. Change in EI among all four Groups (A, B, C, D) before and after UV exposure with and without camouflage, to provide a quantitative assessment of erythema intensity and explore the mechanism of camouflage effects.

#### 2.6.2 Data Presentation

Normally distributed continuous data were expressed as mean ± standard deviation (SD). Non‐normally distributed data were expressed as median (range). The Shapiro–Wilk test was used to assess the normality of distribution.

##### 2.6.2.1 Primary Endpoint Statistical Analysis

The Wilcoxon signed‐rank test (nonparametric paired test) was used to compare erMED values between Group B and D. This test was selected because: (1) subjects served as their own controls, creating paired data; (2) MED values showed non‐normal distribution; and (3) the test provides robust analysis for small sample sizes with ordinal or non‐normally distributed data.

##### 2.6.2.2 Secondary Endpoint Statistical Analyses

1. Validation of measurement methods: The Wilcoxon signed‐rank test was used to compare drMED versus erMED within each group (B and D separately) to assess inter‐method agreement and validate the consistency of visual and dermatoscopic MED assessment.
2. Dermatoscopic MED comparison: The Wilcoxon signed‐rank test compared drMED values between Groups B and D to confirm that findings from visual assessment were reproducible with objective imaging.
3. Erythema Index analysis: Given the four‐group design with all groups tested on each subject, EI analysis employed multiple approaches:
  - Intergroup comparisons: Wilcoxon signed‐rank test with Bonferroni correction for multiple comparisons was applied to compare EI values among all four groups. With six possible pairwise comparisons (A vs. B, A vs. C, A vs. D, B vs. C, B vs. D, C vs. D), the significance threshold was adjusted to α = 0.0083 (0.05/6) to control family‐wise error rate.
  - Background correction: To isolate the specific effect of UV irradiation from background color effects of camouflage, we calculated background‐corrected EI values: EI_B‐A_ = EI_B_−EI_A_ (UV effect without camouflage) and EI_D‐C_ = EI_D_−EI_C_ (UV effect with camouflage). These corrected values were then compared using the Wilcoxon signed‐rank test.
  - Multivariable analysis: Linear regression with interaction effects was performed to comprehensively assess how camouflage, UV radiation, age, gender, and skin type influence EI values. The interaction term (camouflage × UV radiation) specifically tested whether the presence of camouflage modifies the erythema response to UV exposure.

### 2.7 Exploratory Subgroup Analysis

The Mann–Whitney U test (nonparametric independent samples test) was used to compare MED values between Fitzpatrick skin type III and type IV subgroups.

### 2.8 Statistical Significance Criteria

For primary and secondary endpoint analyses, *p* < 0.05 (two‐tailed) was considered statistically significant. For EI comparisons among four groups with Bonferroni correction, *p* < 0.0083 was considered statistically significant.

## 3 Results

### 3.1 Primary Endpoint Results

The primary endpoint—change in refined MED assessed by naked eye (erMED)—demonstrated statistically significant differences between Group B (irradiation without camouflage) and Group D (irradiation with camouflage) (*p* = 0.0047, Wilcoxon signed‐rank test). Group D showed slightly higher MED values compared to Group B, indicating that the presence of DHA camouflage produced a modest photoprotective effect. However, as detailed below, the magnitude of this difference was clinically minimal.

### 3.2 Study Population

A total of 54 healthy volunteers were enlisted and 51 completed the study. The detailed baseline characteristics of the 51 subjects are shown in Table 1. They aged 41 years, with a range of 20 to 62 years. There were 14 (27.5%) males and 37 (72.5%) females. Additionally, 11 (21.6%) of the subjects were skin type III, and the others were skin type IV.

**TABLE 1:** Demographic and clinical characteristics of the study group.

Study population	*n* (%)
Gender	
Male	14 (27.5)
Female	37 (72.5)
Age, years old	41 (20–62)^a^
18–29	12 (23.5)
30–49	20 (39.2)
50–69	19 (37.3)
Skin type	
Type III	11 (21.6)
Type IV	40 (78.4)
Residential location	
Urban	28 (54.9)
Rural	23 (45.1)

^a^ All quantitative data were presented as median (range).

### 3.3 MED After 308 nm Excimer Light Irradiation on the Lower Back of the Subjects

**FIGURE 2:**
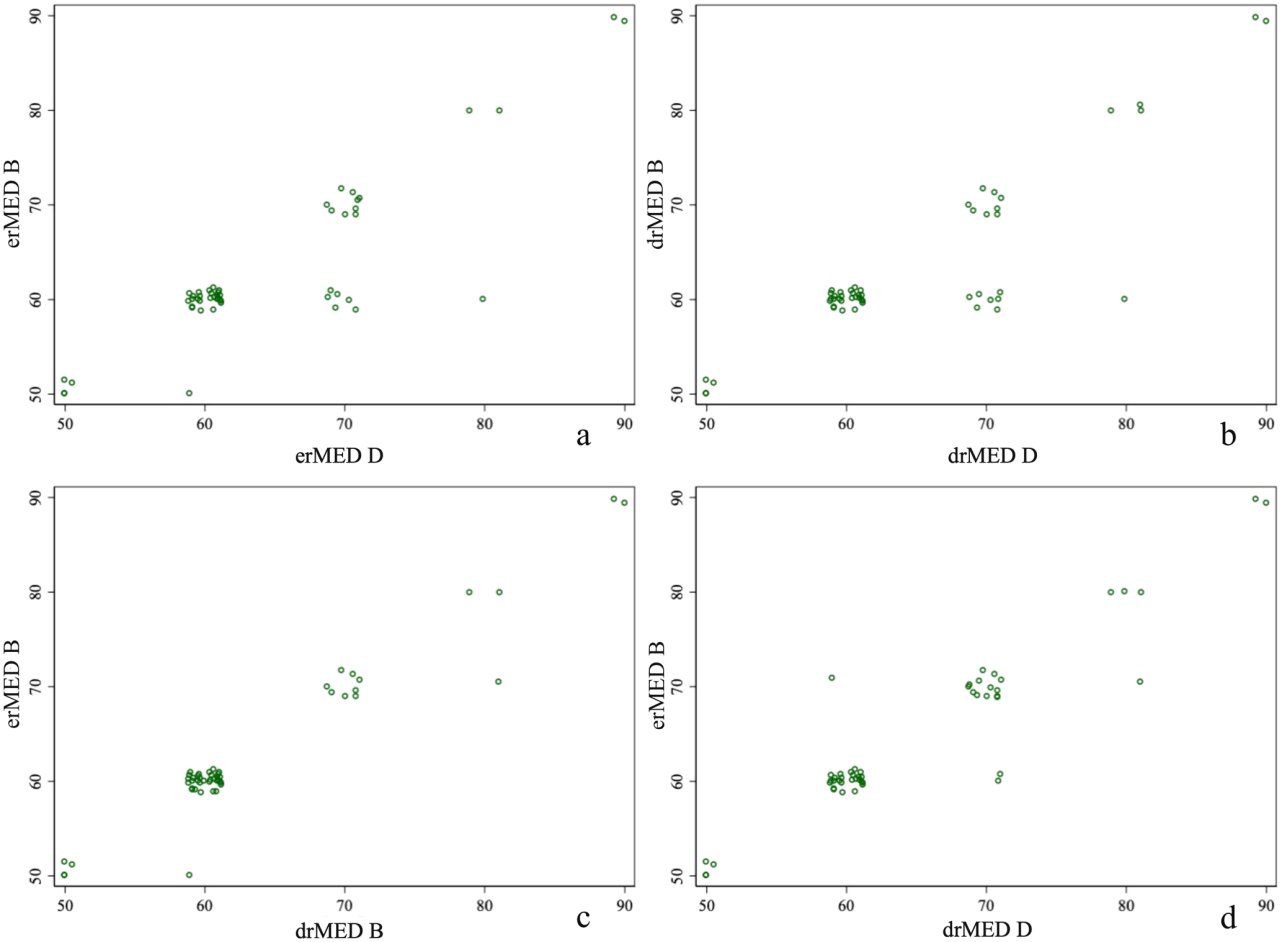
Comparison of MED in irradiated areas (with/without camouflage) by dermatoscopic observation (drMED) and by macroscopic clinical observation (erMED). Comparison between drMED and erMED in Group B (*Z* = 1.414, *p* = 0.157) (a) and Group D (*Z* = 1.000, *p* = 0.317) (b). Comparison erMED and drMED between Group B (*Z* = −2.827, *p* = 0.0047) (c) and Group D (*Z* = −2.827, *p* = 0.0047) (d). drMED, refined MED observed using a digital dermatoscope (Foto Finder handyscope); erMED, refined MED observed by naked eyes.

**FIGURE 3:**
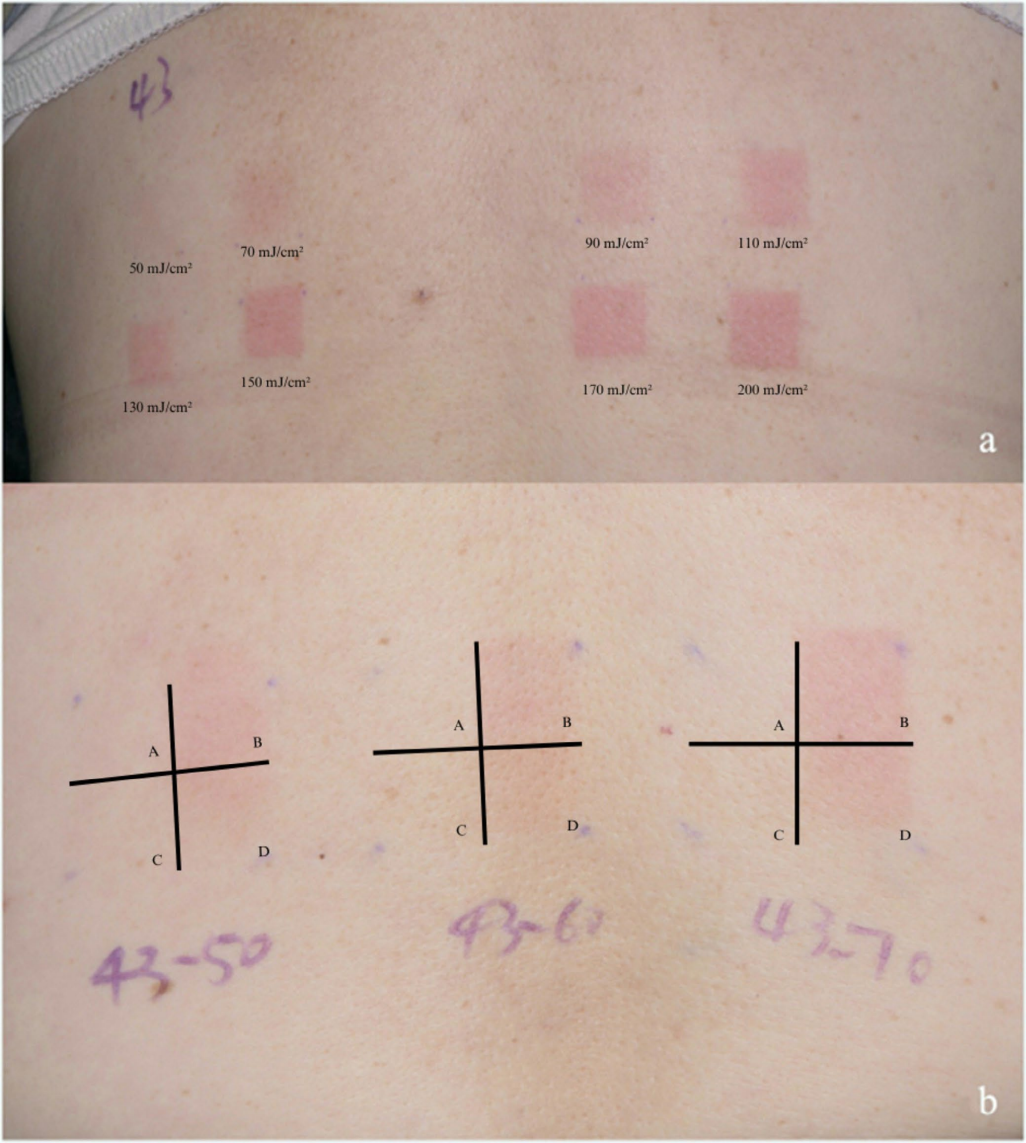
Macroscopic erythema on the lower back of a subject after irradiation. Pictures of a patient (#43). (a) cMED determination showing erythema response to different UV doses (50–200 mJ/cm^2^ as labeled). (b) rMED test on the lower back with three test areas irradiated at 50, 60, and 70 mJ/cm^2^, each area divided into four quadrants labeled A, B, C, D representing the four experimental groups: Group A, no camouflage nor irradiation; Group B, irradiation without camouflage; Group C, camouflage without irradiation; Group D, camouflage plus irradiation.

**FIGURE 4:**
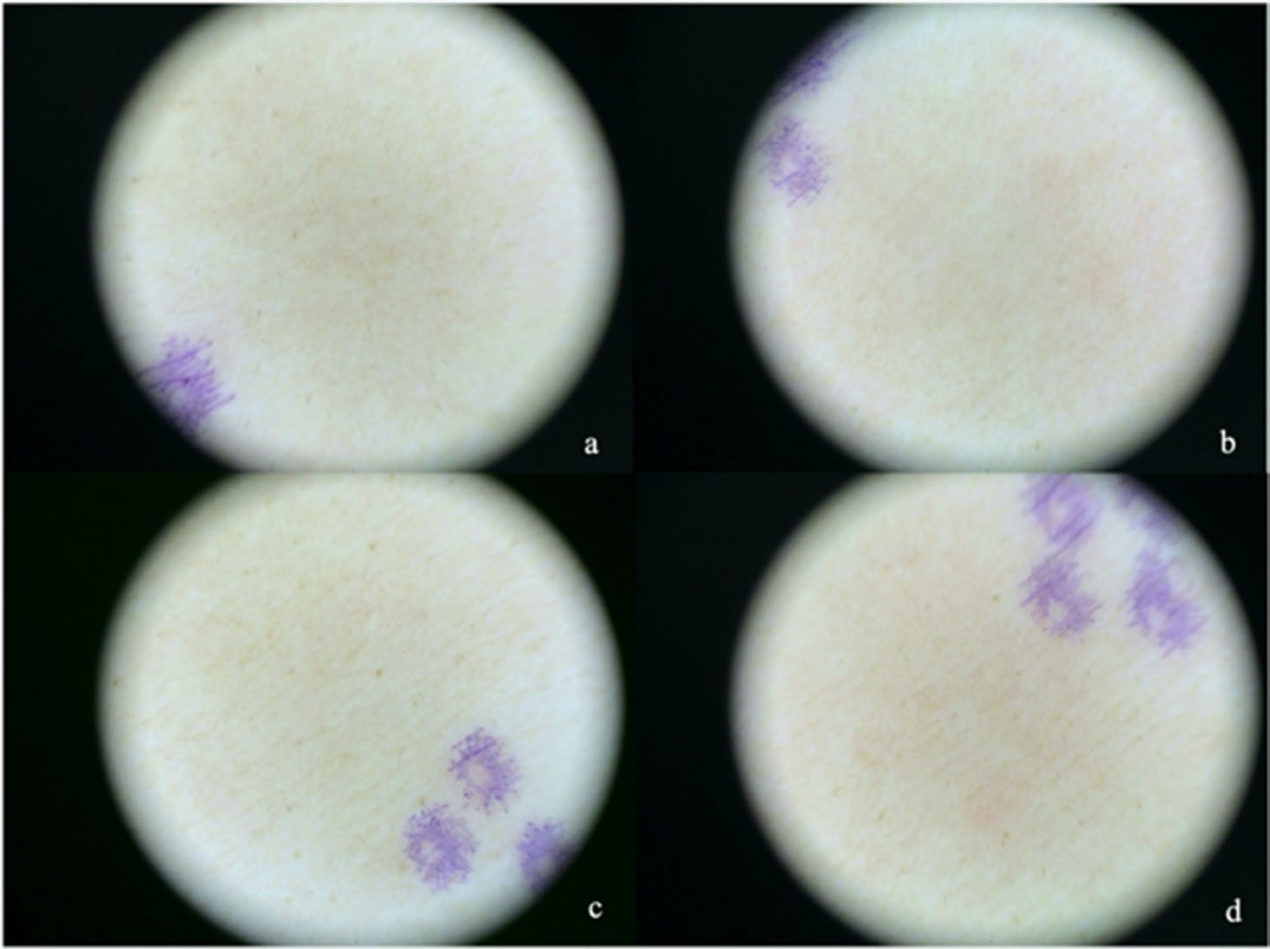
Dermatoscopic erythema on the lower back of a subject after irradiation. Pictures of a patient (#43). The patient had erythema observed under the dermatoscope after irradiation with MED. The erythema was analyzed by IMAGE J, and the EI values were 8.37, 10.44, 9.40, and 12.22 for groups A, B, C, and D, respectively [a, DHA (−) UV (−); b, DHA (−) UV (+); c, DHA (+) UV (−); and d, DHA (+) UV (+)].

All the 51 subjects had an average cMED of 70 (50–110) mJ/cm^2^. Subjects in Group B represented the MED tested without camouflage, and the average erMED was 60 (50–90) mJ/cm^2^ (vs. drMED, 60 (50–90) mJ/cm^2^, *p* = 0.157, Table 2, Figure 2a). Subjects in Group D represented the MED tested after camouflage, and the average erMED was 60 (50–90) mJ/cm^2^ (vs. drMED, 60 (50–90) mJ/cm^2^, *p* = 0.317, Figure 2b). There was a statistically significant difference in erMED between Group B and D (*p* = 0.0047, Figure 2c). Similarly, there was a statistically significant difference in drMED between Group B and D (*p* = 0.0047, Table 3, Figure 2d). An example of cMED determination with dose labeling (Figure 3a), rMED test layout with experimental groups (Figure 3b), and drMED evaluation (Figure 4) is shown. A total of 43 remaining subjects (84.3%) had unchanged erMED after the camouflage masking, and 7 (13.7%) had a MED with an increase of one grade (+10 mJ/cm^2^), and only one subject (2.0%) had a MED increase of two grades (+20 mJ/cm^2^).

**TABLE 2:** Comparison of MED in irradiated areas by dermatoscopic observation (drMED) and by macroscopic clinical observation (erMED).

drMED (mJ/cm^2^)	erMED (mJ/cm^2^)											
	Group B						Group D					
	50	60	70	80	90	Total	50	60	70	80	90	Total
50	4	0	0	0	0	4	4	0	0	0	0	4
60	1	33	0	0	0	34	0	25	1	0	0	26
70	0	0	8	0	0	8	0	2	13	0	0	15
80	0	0	1	2	0	3	0	0	1	3	0	4
90	0	0	0	0	2	2	0	0	0	0	2	2
Total	5	33	9	2	2	51	4	27	15	3	2	51

*Note:* Comparison between drMED and erMED in Group B (*Z* = 1.414, *p* = 0.157) and in Group D (*Z* = 1.000, *p* = 0.317).

Abbreviations: drMED, refined MED observed using a digital dermatoscope (Foto Finder handyscope); erMED, refined MED observed by naked eyes.

**TABLE 3:** MED changes after irradiation with or without camouflage.

MED without camouflage	MED increase when covered with camouflage						Total number of subjects	
	0 mJ/cm^2^		10 mJ/cm^2^		20 mJ/cm^2^			
	erMED	drMED	erMED	drMED	erMED	drMED	erMED	drMED
50 mJ/cm^2^	4 (7.8)	4 (7.8)	1 (2.0)	0 (0.0)	0 (0.0)	0 (0.0)	5 (9.8)	4 (7.8)
60 mJ/cm^2^	26 (51.0)	26 (51.0)	6 (11.8)	7 (13.7)	1 (2.0)	1 (2.0)	33 (64.7)	34 (66.7)
70 mJ/cm^2^	9 (17.6)	8 (15.7)	0 (0.0)	0 (0.0)	0 (0.0)	0 (0.0)	9 (17.6)	8 (15.7)
80 mJ/cm^2^	2 (3.9)	3 (5.9)	0 (0.0)	0 (0.0)	0 (0.0)	0 (0.0)	2 (3.9)	3 (5.9)
90 mJ/cm^2^	2 (3.9)	2 (3.9)	0 (0.0)	0 (0.0)	0 (0.0)	0 (0.0)	2 (3.9)	2 (3.9)
Total	43 (84.3)	43 (84.3)	7 (13.7)	7 (13.7)	1 (2.0)	1 (2.0)	51 (100.0)	51 (100.0)

*Note:* All data in the table are number with percentage. Comparison erMEDs between Group B and D (*Z* = −2.827, *p* = 0.0047) and drMEDs between Group B and D (*Z* = −2.827, *p* = 0.0047).

Abbreviations: drMED, refined MED observed using a digital dermatoscope (Foto Finder handyscope); erMED, refined MED observed by naked eyes.

### 3.4 Effect of Camouflage Agent on EI

**FIGURE 5:**
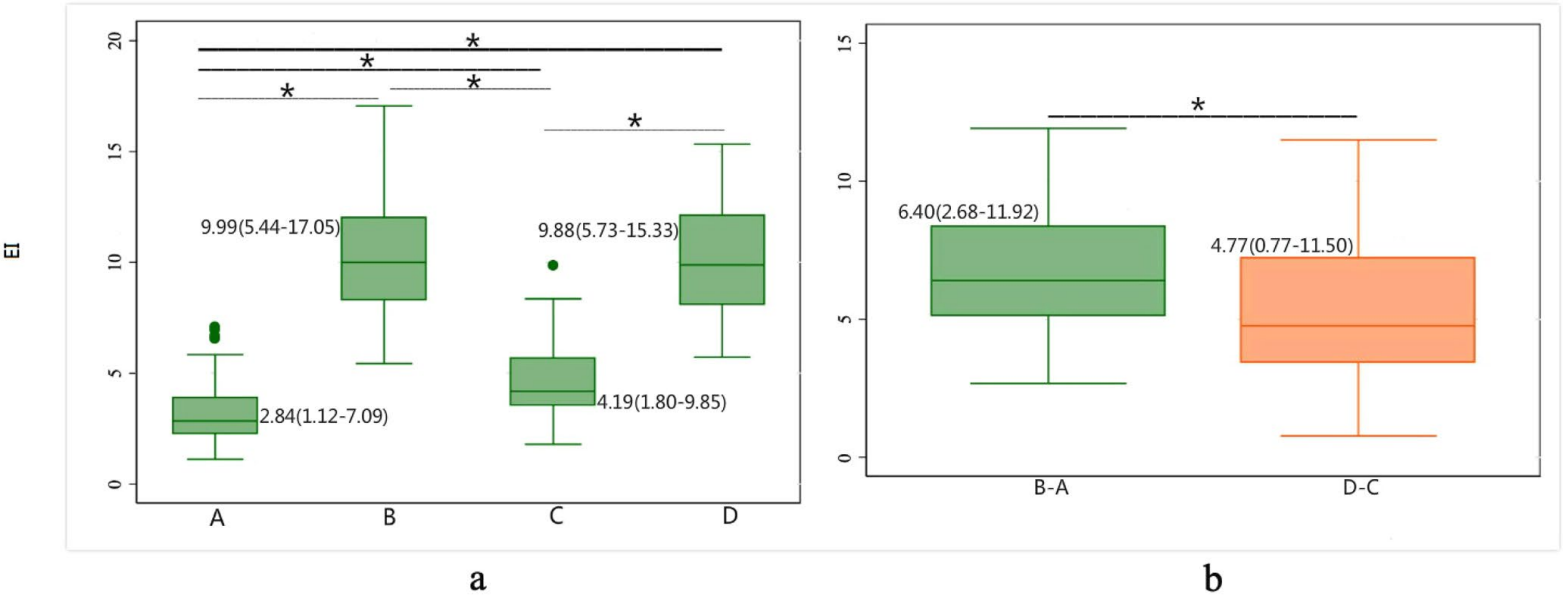
Erythema index (EI) with/without 308 nm light irradiation at the dose of one MED. Three 4 × 4 cm squares were outlined at the intended irradiation sites and equally divided into four parts (as shown in Figure S2). Each part corresponded to one of the groups: Group A, no camouflage nor irradiation; Group B, irradiation without camouflage; Group C, camouflage without irradiation; Group D, camouflage plus irradiation. The Erythema index (EI) of the irradiated area was analyzed by examining the dermoscopy images of the targeted sites via Image J software. Wilcoxon signed‐rank test with Bonferroni correction was used to compare the difference of EI between Groups A, B, C, and D (a) and the difference after controlling the effect of background on EI (EIB‐A and EID‐C) (b). A significant difference was found between Group A and B (*Z* = −6.215, *p* = 0.000), Group C and D (*Z* = −6.215, *p* = 0.000), Group A and C (*Z* = −6.215, *p* = 0.000), Group EIB‐A and EID‐C (*Z* = 3.173, *p* = 0.0015), but not between Group B and D (*Z* = −0.103, *p* = 0.918). drMED, refined MED observed using a digital dermatoscope; EI, erythema index obtaining special images from ordinary digital color images using ImageJ freeware to analyze the change of erythema; EIB‐A, The difference of EI between Group B and A; EID‐C, The difference of EI between Group D and C; erMED, refined MED observed by naked eyes. *p < 0.05.

By analyzing the dermatoscopic images, Group A was estimated with an EI of 2.844 (1.116–7.092). The EI was 9.994 (5.436–17.054) in Group B, 4.187 (1.795–9.846) in Group C, and 9.882 (5.727–15.331) in Group D (Figure 5a). Wilcoxon signed‐rank test with Bonferroni correction analysis showed that there was a significant difference between Group A and C (*p* = 0.000), indicating that the camouflage agent affected EI. A significant difference was found in EIs between Group A and B (*p* = 0.000), as well as Group C and D (*p* = 0.000), but not between Group B and D (*p* = 0.918, Figure 5a). After controlling for the EI effect of the background, the EI of irradiation without camouflage (EIB‐A) was 6.400 (0.777–11.499), and the EI of irradiation with camouflage (EID‐C) was 4.765 (2.679–11.919) (Figure 5b). There was a significant difference between Group EIB‐A and EID‐C (*p* = 0.0015, Figure 5b).

### 3.5 Biological Interpretation of Baseline EI Elevation

Notably, camouflage application alone (Group C) significantly increased EI values compared to untreated skin (Group A) (*p* = 0.000), despite the complete absence of UV irradiation and therefore no biological basis for genuine erythema. Group C exhibited a median EI of 4.187 compared to 2.844 in Group A—representing an approximately 47% increase. This substantial EI elevation in the absence of any inflammatory stimulus strongly suggests that DHA‐induced pigmentation creates optical interference with the image analysis algorithm's red channel detection, rather than indicating true vascular or inflammatory changes. The melanoidins produced by DHA‐keratin reactions absorb light across a broad spectrum and may alter the skin's reflectance properties in ways that are interpreted by digital image analysis software (ImageJ) as increased “redness,” even when no vascular dilation, inflammatory cell infiltration, or erythema mediator release has occurred.

### 3.6 Linear Regression Analysis With Interaction Effects of the Effects of Factors on EI Value

Camouflage, UV radiation, camouflage plus UV radiation, and age were the independent factors contributing to final EI values (*p* < 0.05), while gender and skin type were irrelevant to EI. The camouflage alone (without UV radiation) can slightly increase the EI value (*p* = 0.001, Table S1). There was no significant difference in EI values between camouflage and camouflage combined with 1 MED UV radiation (*p* = 0.918, Figure 5a).

## 4 Discussion

Vitiligo has a great social‐psychological burden on the patients. Currently, there is no cure for vitiligo; therefore, the appropriate use of cosmetics to cover vitiligo lesions is considered beneficial for enhancing patients' quality of life [11] and is recommended by guidelines [12]. Camouflage products containing 3.6% DHA have been widely used for vitiligo lesion coverage. This kind of camouflage can remain on the skin for a period of as long as 1 week. Due to a lack of research, there are no specific guidelines on the use of DHA‐containing camouflage agents during phototherapy for patients [13].

The study design of applying camouflage before UV irradiation reflects real clinical scenarios. DHA‐containing camouflage agents bind firmly to epidermal keratin and can persist for 7–15 days, making complete removal difficult even with regular cleansing. Many vitiligo patients prefer to continue phototherapy without completely removing their camouflage for psychological and social reasons. Therefore, our study design addresses a practical clinical question: whether phototherapy can be safely and effectively performed in the presence of camouflage agents.

### 4.1 Literature Reconciliation and Clinical Significance

Previous studies have reported photoprotective effects of DHA‐containing products. Faurschou and Wulf [14] applied 10% DHA cream twice daily, observing SPF values of 3.0, 2.0, and 1.7 after 1, 5, and 7 days, respectively. Similarly, studies using 5% or 20% DHA formulations reported SPF increases of 1.3 to 2.2 [15]. In contrast, our study examined a 3.6% DHA formulation—the clinically relevant concentration used in commercially available long‐lasting camouflage products. The relationship between DHA concentration and UV photoprotection is dose‐dependent, as higher concentrations produce greater quantities of melanoidin chromophores. Our lower DHA concentration provides a plausible explanation for the substantially reduced photoprotective effect observed.

Standard SPF measurements employ broad‐spectrum UVB radiation (290–320 nm), while our study specifically examined monochromatic 308 nm excimer light—a wavelength at the long‐wave end of the UVB spectrum. At 308 nm, melanoidin absorption may be substantially lower than at shorter, more erythemagenic wavelengths (295–305 nm) where traditional SPF testing is most sensitive. Additionally, Faurschou et al. employed repeated applications twice daily, potentially creating progressively thicker pigmented layers, whereas our single application represents steady‐state pigmentation.

Quantitative analysis shows our mean photoprotection is substantially lower than SPF 1.3, consistent with lower DHA concentration (3.6% vs. 5%–20%) and potentially reduced absorption at 308 nm. Our findings suggest that at the 308 nm wavelength and 3.6% DHA concentration, the photoprotective effect is insufficient to contraindicate concurrent phototherapy. Even previously reported SPF 1.3–3 values translate to manageable MED increases within standard phototherapy protocols.

Critically, distinguishing statistical from clinical significance is essential. While statistical analysis revealed significant differences in both MED (*p* = 0.0047) and EI values (*p* = 0.0015) between Groups B (irradiation without camouflage) and D (irradiation with camouflage), the clinical impact is minimal. The magnitude of MED change (0–20 mJ/cm^2^) represents only 0%–33% of baseline MED values and is substantially smaller than typical dose‐escalation steps used in clinical phototherapy protocols (usually 10%–20% of the previous dose or 50–100 mJ/cm^2^ increments). Most importantly, 84.3% of subjects required no dose adjustment whatsoever, and the remaining 15.7% needed only minimal increases (10–20 mJ/cm^2^) easily managed within standard protocols. Therefore, while DHA camouflage has a measurable, statistically significant effect on UV‐skin interaction, this effect does not pose a clinically significant barrier to effective 308‐nm excimer therapy. In most cases, phototherapy can proceed normally without adjusting the radiation dose.

### 4.2 MED Variability and Measurement Considerations

MED test is frequently utilized in clinical settings to determine the minimum amount of ultraviolet (UV) exposure required to produce erythema (inflammatory redness) on the skin surface. Additionally, the MED test has the potential to serve as a robust tool for assessing changes in the inflammatory response of individual subjects or population [16]. Our study showed that the MED on the back of 51 healthy volunteers [60 (50–90) mJ/cm^2^] was lower than in previous studies, ranging from 114.05–350 mJ/cm^2^ [17, 18]. MED may be influenced by various factors, including the light source, exposure distance, detection methods, seasonality, meteorological conditions, skin type, gender, previous UV exposure, and anatomical characteristics [19]. In this study, the irradiation device, exposure distance, irradiation sites, and the population were different from previous studies, which might contribute to the relatively lower MED.

### 4.3 Optical Interference in Erythema Assessment

Our findings reveal an important methodological consideration: DHA camouflage appears to create optical interference with digital erythema detection rather than biologically modifying the inflammatory response. Supporting evidence includes:

1. EI elevation without inflammation: Camouflage alone (Group C) significantly elevated EI values by approximately 47% compared to untreated skin (Group A) despite the complete absence of UV exposure. Since no inflammatory stimulus was present, this EI elevation cannot represent true erythema, demonstrating that DHA pigmentation itself artifactually increases calculated EI values.
2. Discordance between EI and clinical MED: While EI showed significant differences in background‐corrected analysis (*p* = 0.0015), clinical MED assessment revealed 84.3% of subjects had unchanged erMED. This marked discordance suggests that EI changes predominantly reflect measurement artifact rather than true differences in inflammatory response.
3. Mechanistic basis: DHA‐derived melanoidins are brown chromophores with broad‐spectrum light absorption that may partially absorb or scatter red wavelength light (550–650 nm) used for erythema quantification, alter overall color balance in digital images, and create spectral confusion in RGB‐based algorithms not designed for artificial pigmentation.
4. Clinical implications: These findings underscore that clinical MED determination based on visible inflammatory borders must remain the gold standard for phototherapy dosing decisions. While digital dermatoscopy remains valuable for documentation, quantitative colorimetric indices may be unreliable in pigmented areas. The strong correlation between erMED and drMED (*p* = 0.157 and *p* = 0.317) demonstrates that structural erythema detection remains valid using morphological features rather than color indices. This optical interference phenomenon has implications for assessing erythema in naturally darker skin types, where constitutive melanin may similarly interfere with RGB‐based detection algorithms.

### 4.4 Application Protocol: Single vs. Chronic Use

Our study design—single camouflage application 24 h before UV exposure—warrants interpretation regarding chronic use:

1. Pharmacokinetic rationale: The DHA‐keratin reaction is largely complete within 24 h, forming stable melanoidins that do not undergo further modification. Unlike cumulative drugs, each application creates a discrete pigmentation layer that desquamates with the stratum corneum (7–15 day half‐life). Our single‐application model at 24 h effectively represents steady‐state conditions.
2. Potential cumulative effects and clinical context: Chronic repeated applications could theoretically result in thicker pigmented layers if applications occur before complete desquamation, deeper keratin penetration, or altered barrier function affecting UV penetration. These effects, if present, would likely manifest as enhanced photoprotection requiring slightly greater dose adjustments. However, in practice, vitiligo patients typically apply camouflage weekly or less frequently, allowing substantial stratum corneum turnover. Our findings of minimal photoprotection (84.3% requiring no adjustment) suggest that even if cumulative effects exist, they remain clinically manageable.
3. Future research priorities: Prospective studies examining patients undergoing repeated applications over 8–12 week phototherapy courses would validate our findings and determine whether dose algorithms require modification for chronic users.

### 4.5 Study Limitations and Future Directions

This study has several important limitations. First, the inclusion of only Fitzpatrick skin types III–IV significantly limits generalizability. This restriction is particularly relevant because:

1. Skin types I–II are more UV‐sensitive with lower baseline MEDs. DHA camouflage might have proportionally greater photoprotective effects on these lighter skin types, potentially requiring larger dose adjustments than observed in our study population.
2. Skin types V–VI have higher constitutive melanin content, which may interact differently with DHA‐induced melanoidins. The optical properties and UV absorption characteristics could differ substantially, potentially altering both photoprotective effects and optical interference with erythema detection.
3. Regional and global relevance: While our findings are directly applicable to East Asian populations (predominantly skin types III–IV), DHA camouflage products are used globally across all skin phototypes.

Second, the relatively small sample size (*n* = 51) limits statistical power for detecting subtle differences. Third, only a single application protocol was tested. Fourth, the study was conducted in a controlled setting, which may not fully reflect real‐world usage patterns where application thickness and frequency vary.

Future investigations should systematically address these limitations through multicenter studies incorporating diverse ethnic populations and all six Fitzpatrick skin types. Priority areas include: (1) examining whether lighter skin types (I–II) require greater dose adjustments due to enhanced photoprotection; (2) investigating whether darker skin types (V–VI) experience different optical interference patterns and whether alternative assessment methods provide more reliable endpoints; (3) elucidating interactions between constitutive and DHA‐induced pigmentation; (4) developing dose adjustment algorithms stratified by skin phototype; and (5) examining repeated applications over complete phototherapy courses to validate chronic use safety and efficacy.

## 5 Conclusion

The camouflage agent containing 3.6% DHA demonstrates statistically significant but clinically negligible effects on the erythemal response induced by a 308 nm excimer lamp in healthy volunteers. In clinical practice, phototherapy can proceed safely in the presence of 3.6% DHA camouflage, with dose adjustments required in fewer than 16% of cases and only minimal increases necessary when adjustments are needed.

## Author Contributions

We declare that all the listed authors have participated actively in the study and all meet the requirements of authorship. D.S. and Y.H. designed the study and wrote the paper. Z.L. managed the data acquisition. D.C. undertook the data analysis. Y.Z. contributed to the correspondence and paper revision. All authors reviewed the manuscript.

## Funding

This study is funded by the Beijing Hospitals Authority's Ascent Plan, Code: DFL20240901. National Key Research and Development Program of China 2023YFC2508100.

## Ethics Statement

The study was approved by the Ethics Committee of the Beijing Tsinghua Changgung Hospital, and it is in accordance with the Declaration of Helsinki.

## Consent

All participants signed informed consent forms.

## Conflicts of Interest

The authors declare no conflicts of interest.

## Supporting information


**Figure S1:** Graphical description of the order and doses for MED rough measurement.
**Figure S2:** Observation of the influence of the camouflage agent on MED. (A) Group DHA (−) UV (−); (B) Group DHA (−) UV (+); (C) DHA (+) UV (−); and (D) DHA (+) UV (+). (color: with camouflage agent DHA; dot: with irradiation).
**Table S1:** Linear regression with interaction effects was used to study the effects of factors on EI value.

## Data Availability

The datasets used or analyzed during the current study are available from the corresponding author on reasonable request.
