# Supplementary material for: Effect of a Dihydroxyacetone‐Based Camouflage Agent on Ultraviolet‐Induced Erythema
Source: J Cosmet Dermatol. 2025 Dec 5;24(12):e70587. doi: 10.1111/jocd.70587 (PMC12679678; doi:10.1111/jocd.70587)
Supplement: Supplementary file 1 — Figure S1: Graphical description of the order and doses for MED rough measurement. Figure S2: Observation of the influence of the camouflage agent on MED. (A) Group DHA (−) UV (−); (B) Group DHA (−) UV (+); (C) DHA (+) UV (−); and (D) DHA (+) UV (+). (color: with camouflage agent DHA; dot: with irradiation). Table S1: Linear regression with interaction effects was used to study the effects of factors on EI value. [file JOCD-24-e70587-s001.docx]

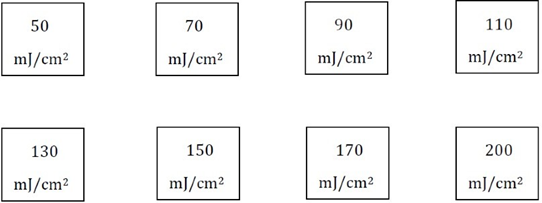


**Figure S1. Graphical description of the order and doses for MED rough measurement.**


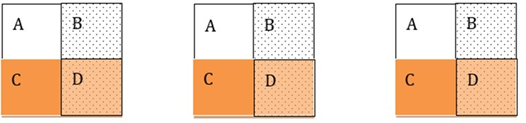


**Figure S2. Observation of the influence of the camouflage agent on MED.** (A) group DHA (-) UV (-); (B) group DHA (-) UV (+); (C) DHA (+) UV (-); and (D) DHA (+) UV (+). (color: with camouflage agent DHA; dot: with irradiation).

**Table S1. Linear regression with interaction effects was used to study the effects of factors on EI value.**

| ei | Coef. | Std. Err. | *t* | *p*>\|t\| | 95% Conf. Interval | |
| --- | --- | --- | --- | --- | --- | --- |
| no | .00939 | .01040 | 0.90 | 0.367 | -0.01112 | 0.02991 |
| _Iuv_1 | 6.8173 | .42982 | 15.86 | 0.000 | 5.96964 | 7.66499 |
| _Icamouflag_1 | 1.51270 | .42982 | 3.52 | 0.001 | 0.66503 | 2.36038 |
| _IuvXcam_1_1 | -1.54015 | .60786 | -2.53 | 0.012 | -2.73895 | -.34136 |
| _Igender_1 | 1.06255 | .35569 | 2.99 | 0.003 | .36107 | 1.76404 |
| age | .00186 | .01331 | 0.14 | 0.889 | -.02440 | .02811 |
| _Istype_4 | .31302 | .40087 | 0.78 | 0.436 | -.47757 | 1.10360 |
| _cons | 2.41094 | .62332 | 3.87 | 0.000 | 1.18167 | 3.64023 |

Camouflage, UV radiation, camouflage plus UV radiation and age were independent factors on EI values (*p*<0.05) while sex and skin type were irrelevant with EI. The camouflage alone (without UV radiation) can slightly increase the EI value (*p*=0.001).
